# Artificial intelligence in the operating room: A systematic review of AI models for surgical phase, instruments and anatomical structure identification

**DOI:** 10.1111/aogs.70045

**Published:** 2025-08-27

**Authors:** Sara Paracchini, Cristina Taliento, Giulia Pellecchia, Veronica Tius, Madalena Tavares, Chiara Borghi, Alessandro Antonio Buda, Adrien Bartoli, Nicolas Bourdel, Giuseppe Vizzielli

**Affiliations:** ^1^ Gynecology Oncology Surgical Unit, Department of Obstetrics and Gynecology Ospedale Michele e Pietro Ferrero Verduno Italy; ^2^ Department of Medicine (DMED), University of Udine Udine Italy; ^3^ Department of Medical Sciences University of Ferrara Ferrara Italy; ^4^ Clinic of Obstetrics and Gynecology, “S. Maria Della Misericordia” University Hospital, Azienda Sanitaria Universitaria Friuli Centrale (ASUFC) Udine Italy; ^5^ Department of Gynecology Hospital Da Luz Lisbon Portugal; ^6^ EnCoV, Institut Pascal, UMR 6602, CNRS/UCA Clermont‐Ferrand France; ^7^ SURGAR, Surgical Augmented Reality Clermont‐Ferrand France; ^8^ Department of Clinical Research and Innovation Clermont‐Ferrand University Hospital Clermont‐Ferrand France; ^9^ Department of Obstetrics and Gynecology University Hospital Clermont‐Ferrand Clermont‐Ferrand France

**Keywords:** artificial intelligence, instrument recognition, minimally invasive surgery, surgical step recognition

## Abstract

**Introduction:**

This systematic review examines the application of multiple deep learning algorithms in the analysis of intraoperative videos to enable feature extraction and pattern recognition of surgical phases, anatomical structures, and surgical instruments.

**Material and Methods:**

A comprehensive literature search was conducted across PubMed, Web of Science, and EBSCO, covering studies published until March 2024. This review includes studies that applied AI models in the operating room for surgical‐phase recognition and/or anatomical structures and instruments. Only studies utilizing machine learning or deep learning for surgical video analysis were considered. The primary outcome measures were accuracy, precision, recall, and F1 score.

**Results:**

A total of 21 studies were included. Multilayer architecture of interconnected neural networks was predominantly used. The deep learning models demonstrated promising results, with accuracy ranging from 81% to 93.2% for surgical‐phase recognition. Anatomical structure recognition models achieved accuracy between 71.4% and 98.1%.

**Conclusions:**

Artificial intelligence has the potential to significantly improve surgical precision and workflow, with demonstrated success in phase recognition and anatomical structure identification. However, further research is needed to address dataset limitations, standardize annotation protocols, and minimize biases.

## Graphical abstract

Deep learning models applied to surgical videos enable accurate recognition of phases, instruments, and anatomical structures. This technology enhances intraoperative guidance, improves patient safety, and supports surgical education.

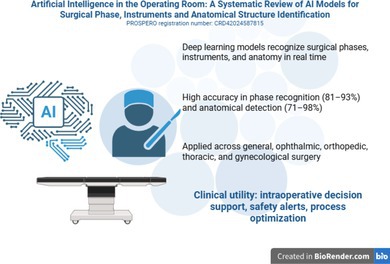

**Key message**

Deep learning exhibits high accuracy in surgical phases, anatomical structures, and surgical instruments.

## 1 INTRODUCTION

The advent of minimally invasive surgery has led to a large and increasingly growing amount of valuable digital data extracted from procedural videos recorded during surgery in the operating rooms. Development and integration of artificial intelligence (AI) into the surgical workflow hold great promise for the future of surgery. On a larger scale, video recording and analysis by intelligent systems could help monitor the surgical processes: they could provide automated assistance at the operating table in phase recognition, tools, and anatomical landmark detection, alerting surgeons about risks and complications by identifying intraoperative safety milestones (ureters and major vessels) and adverse events (severe bleeding).^1^, ^2^

Surgical step detection is the process of dividing surgical video into landmark steps and predefined actions, transforming procedural videos into segmented video in which classification algorithms based on AI are employed for detecting these predefined blocks, i.e., surgical actions or anatomical structures.^3^ The emerging field of surgical‐phase recognition relies on deep learning, which is a machine learning technique based on artificial neural networks capable of feature extraction and recognition.

Deep learning has already been extensively applied to enhance medical image analysis, such as for skin cancer classification and diagnostic accuracy of several pathologies.^4^, ^5^, ^6^ Recently, this technology has been applied in the surgical domain, especially in routine and standardized endoscopic procedures.

This study assesses whether deep learning applications can accurately analyze surgical videos across various surgical specialties. To address this question, we conducted a systematic review of available deep learning and automated video analysis tools, focusing on the recognition and detection of surgical phases, instruments, actions, and anatomical structures. From a broader perspective, this study critically examines the emerging evidence within the field of AI in gynecological surgery and outlines future directions for research and clinical application. The systematic review stems from the need to fine‐tune the state of the art of scientific evidence in an organized manner on the role of deep learning in the operating room as a support to the surgeon and for training in the early learning of surgical steps and surgical instruments. Specifically, the primary aim of this systematic review is to assess the reported applications and advancements in deep learning for intraoperative video recording and analysis, including the automated assessment of surgical phases, as well as the detection of instruments and anatomical structures. Secondly, it aims to critically frame the emerging evidence within the context of AI in gynecological surgery and to outline future perspectives.

## 2 MATERIAL AND METHODS

Studies that described the application of AI models in the operating room for the recognition of surgical steps, surgical instruments, or anatomical structures were considered eligible for systematic review regardless of the surgical field considered. Letters to editors, narrative reviews on the subject, and articles not written in English were not eligible for inclusion.

### 2.1 Eligibility criteria

We included studies that applied AI models in the operating room to distinguish phases of a surgical procedure and/or identify anatomical structures during an intervention. Only studies using machine learning or deep learning techniques for real‐time or retrospective analysis of surgical videos were considered. No restrictions were placed on the type of surgery or the AI model architecture used. Both retrospective and prospective studies were eligible, provided they offered data on AI performance metrics such as accuracy, precision, recall, or F1 score.

### 2.2 Information sources and search strategy

A comprehensive search of the literature was conducted across three different databases, including PubMed, Web of Sciences, and EBSCO. We included studies published up to March 2024. The search strategy included combinations of keywords and Medical Subject Headings (MeSH) terms related to AI, machine learning, deep learning, surgery, phase recognition, and anatomical structure identification. Additionally, reference lists of included studies were manually screened to identify any additional publications. The complete search strategy can be found in the Supporting Information Appendix S1. PROSPERO registration number: CRD42024587815.

### 2.3 Study selection

The study selection process was carried out in two stages. First, titles and abstracts of the identified articles were screened independently by two authors (GP and VT) to exclude irrelevant studies. In the second stage, the full text of the remaining articles was assessed for eligibility based on the inclusion criteria. Any disagreements between the two authors were resolved through discussion with a third and fourth reviewer (CT and SP).

### 2.4 Data extraction

Data extraction was performed independently by two reviewers (GP and VT) using a standardized data extraction sheet. The following data were collected for each study: study year, type of study, country, type of surgery, AI model, equipment, surgical procedure, intervention, comparator, surgical landmarks, number of videos analyzed, number of surgeons involved in manual video retrieval, and outcome measures. Discrepancies in the data extraction were discussed and resolved by the two reviewers, and any remaining differences were settled by consulting a third author (SP).

### 2.5 Assessment of risk of bias

One author (SP) independently assessed the risk of bias of the included studies using the New‐Castle Ottawa Scale (NOS) Risk of Bias assessment tool,^7^ which rates studies from 1 to 9 based on selection criteria, comparability for confounding factors, and outcome assessment, with 9 indicating the highest quality. Assessment of sufficiency and adequacy of follow‐up was not applicable in this context due to the design of included studies.

### 2.6 Data synthesis

Data were synthesized using a narrative approach, focusing on the performance of AI models across different types of surgery and their ability to identify surgical phases and anatomical structures. Quantitative data, such as accuracy, F1 scores, and recall, were reported where available. Due to the heterogeneity of the included studies in terms of surgery type, AI model, and outcome measures, a meta‐analysis was not conducted.

## 3 RESULTS

### 3.1 Study selection and characteristics

**FIGURE 1:**
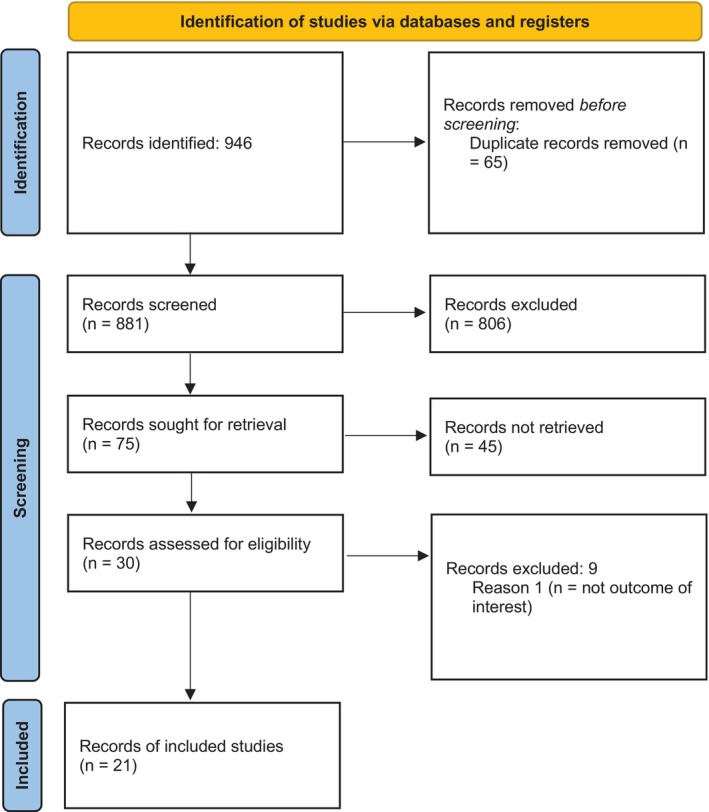
PRISMA Flow‐Chart for the selection process.

Initially, 946 records were identified through database and register searches. Before screening, 65 duplicate records were removed, leaving 881 records to be screened; this resulted in 75 reports sought for retrieval. Subsequently, 30 reports were assessed for eligibility, and 9 of them were excluded because they did not meet the outcome of interest. Finally, a total of 21 studies^8^, ^9^, ^10^, ^11^, ^12^, ^13^, ^14^, ^15^, ^16^, ^17^, ^18^, ^19^, ^20^, ^21^, ^22^, ^23^, ^24^, ^25^, ^26^, ^27^, ^28^ were included in this review, focusing on the application of AI models to recognize surgical phases and/or anatomical structures in various surgical procedures, including general surgery, orthopedic, ophthalmology, and thoracic surgery, and applying a variety of AI models. Figure 1 shows the PRISMA Flow‐Chart for the selection process.

Of the studies included in this review, four^25^, ^26^, ^27^, ^28^ focused on distinguishing anatomical structures, while the remaining studies concentrated on the recognition of surgical phases.

### 3.2 Risk of bias of included studies

NOS risk of bias assessment found mainly low or moderate risk of bias due to possible confounding variables across included studies, indicating overall good quality (Table 1). Main areas of weakness identified were the video selection process and the annotation methodology.

**TABLE 1:** New‐Castle Ottawa Scale Risk of bias assessment.

	Selection				Comparability of cohorts		Outcome			Total
	Representative of the exposed cohort	Selection of external cohort	Ascertainment of exposure	Outcome of interest not present at the start of the study	Main factor	Additional factor	Assessment of outcomes	Sufficient follow‐up time	Adequacy of follow‐up	
Kang, 2024	1	0	1	1	1	0	1	NA	NA	5
Komatsu, 2024	1	1	1	1	1	0	1	NA	NA	6
Fer, 2023	0	0	1	1	1	1	1	NA	NA	5
Ortenzi, 2023	0	1	1	1	1	0	1	NA	NA	5
Golany, 2022	1	0	1	1	1	0	1	NA	NA	5
Kitaguchi, 2022	1	0	1	1	1	0	1	NA	NA	5
Sasaki, 2022	1	0	1	1	1	0	1	NA	NA	5
Takeuchi, 2022	1	0	1	1	1	1	1	NA	NA	6
Kitaguchi, 2020	1	0	1	1	1	0	1	NA	NA	5
Kitaguchi, 2019	0	0	1	1	1	0	1	NA	NA	4
Hashimoto, 2019	0	0	1	1	1	1	1	NA	NA	5
Padoy, 2019	1	1	1	1	1	1	1	NA	NA	7
Yengera, 2018	1	1	1	1	1	1	1	NA	NA	7
Takeuchi, 2022	1	0	1	1	1	0	1	NA	NA	5
Ward, 2021	0	0	1	1	1	0	1	NA	NA	4
Yu, 2019	1	0	1	1	1	1	1	NA	NA	4
Twinanda, 2019	1	0	1	1	1	1	1	NA	NA	6
Smithmaitrie, 2024	1	0	1	1	1	1	1	NA	NA	6
Une, 2024	1	0	1	1	1	1	1	NA	NA	6
Jearanai, 2023	0	0	1	1	1	0	1	NA	NA	4
Mascagni, 2022	0	0	1	1	1	0	1	NA	NA	4

Biases related to video selection include the limited representativeness of the procedures for the broader surgical community and the insufficient diversity of surgical data, despite heterogeneity in surgical images being critical for the development of robust AI models. For instance, some publications focus on highly specific endoscopic procedures with a restricted field of view or no access to the abdominal cavity.^8^, ^11^, ^27^ Annotation‐related biases include limited information about the ground truth labels, poor description of the formal annotation process (e.g., lack of details on the annotators' background, labeling software, and labeling criteria), and the absence of inter‐annotator reliability assessment.^18^ One of the primary sources of bias arises from small and non‐representative datasets. Additionally, many studies do not take into account possible confounding factors, such as the experience level of surgeons or variations in surgical techniques, which can influence phase recognition performance. Furthermore, the lack of standardized protocols for data collection and annotation introduces additional sources of bias, as manual labeling of surgical phases can be subjective and prone to human error. The quality of annotated data is crucial for training deep learning models, as errors in annotation can compromise the reliability and accuracy of the models. Manual annotation of laparoscopic images requires time, expertise, and the involvement of medical professionals, representing one of the current major challenges in deep learning training.

Another methodological limitation highlighted by this review is the heterogeneity in the metrics used to report deep learning performance, which poses a challenge for meaningful comparison across studies.

### 3.3 Surgical‐phase recognition

Data are summarized in Table 2.

**TABLE 2:** Characteristic of studies focusing on Surgical Course.

Study	Type of study	Country	Type of surgery	Dataset	Equipment	Application	Comparator
Orthopedics							
Kang, 2024	OBS	Korea	Orthopedics	Internal and external datasets (educational videos from AAOS)	Keras platform for training implementation	Surgical‐phase recognition Instruments recognition	Manual annotation
General surgery							
Komatsu, 2024	OBS	Japan	General surgery	Proprietary	NVIDIA and an Intel® Xeon® CPU for model training and testing Phyton3.6 for modeling procedures	Surgical‐phase recognition	Manual annotation
Fer, 2023	OBS	USA	General surgery	Proprietary	Internal video annotation tool	Surgical‐phase recognition	Manual annotation
Ortenzi, 2023	OBS	USA Italy	General surgery	Proprietary	Dedicated platform for annotation (not specified)	Surgical‐phase recognition	Manual annotation
Golany 2022	OBS	Israel France	General surgery	Proprietary Cholec80	NA	Surgical‐phase recognition	Manual annotation
Kitaguchi, 2022	OBS	Japan	General surgery	Proprietary ImageNet dataset	NVIDIA and an Intel® Xeon® CPU for model training and testing	Surgical‐phase recognition	Manual annotation
Sasaki, 2022	OBS	Japan	General surgery	Proprietary ImageNet dataset	VISERA ELITE system	Surgical‐phase recognition	Manual annotation
Takeuchi, 2022	OBS	France Japan Rwanda	General surgery	NA	NA	Surgical‐phase recognition	Manual annotation
Kitaguchi, 2020	OBS	Japan	General surgery	Proprietary: LapSig 300	NA	Surgical‐phase recognition Instrument recognition Action ewcognition	Manual annotation
Kitaguchi 2019	OBS	Japan	General surgery	Proprietary: LAP‐S	VISERA ELITE system and ENDOEYE 2D endoscope	Surgical‐phase recognition Action recognition	Manual annotation
Hashimoto, 2019	OBS	USA	General surgery	Proprietary: SleeveNet	NVIDIA, Titan XP GPU The Anvil Video Annotation Research Tool for annotation	Surgical‐phase recognition	Manual annotation
Padoy, 2019	REV	France	General surgery	Cholec120	CAMMA surg flow Live	Surgical‐phase recognition	Manual annotation
Yengera, 2018	REV	France	General surgery	Cholec120	NA	Surgical‐phase recognition	Manual annotation
Thoracic surgery							
Takeuchi 2022	OBS	Japan	Thoracic surgery		NA	Surgical‐phase recognition Phase duration recognition	Manual annotation
Neurology							
Ward, 2021	OBS	USA Japan	Neurology Gastroenterology	Proprietary: POEMNet	FFmpeg software for video de‐identification	Surgical‐phase recognition	Manual annotation
Ophtalmology							
Yu, 2019	OBS	USA	Ophtalmology	SqueezeNet	Python version 3.6 to implement the SVM, RNN and CNN	Surgical‐phase recognition Instruments recognition Evaluation of different machine learning algorithms to classify a video's segment	Manual annotation
Gastroenterology							
Twinanda, 2019	REV	France	Gastroenterology	Cholec120 and Bypass170 datasets	Endo‐net for phase recognition and tool presence detection	Phase duration recognition	

Abbreviations: AI, artificial intelligence; CNN, convolutional neural network; CV, computer vision; CVS, critical view of safety; GPU, graphic processing unit; HMM, hidden Markov models; LSTM, long short‐term memory; ML, machine learning; MS‐TCN, multi‐stage – temporal convolutional network; OBS, observational study; REV, review article; RNN, recurrent neural network; SA, surgical action; SD, surgical duration; SI, surgical instruments; SPR, surgical‐phase recognition; SVV, support vector machine; USA, United States of America; VTN, video transformer network.

#### 3.3.1 General surgery

In 12 studies, convolutional neural networks (CNNs) were employed to recognize surgical phases in general surgery procedures. For instance, Fer et al.^10^ used a multi‐stage temporal convolutional network (MS‐TCN) with ResNet and achieved an F1 score of over 90% in identifying 7 out of 12 steps during Roux‐en‐Y Gastric Bypass procedures. Similarly, Kitaguchi et al.^16^ employed the Xception model to classify phases in colorectal surgery, achieving an overall accuracy of 81.0% for phase recognition. Another study by Ortenzi et al.^11^ applied a Long Short‐Term Memory (LSTM) network to inguinal hernia repairs, obtaining an overall accuracy of 88.8% with the highest accuracy for the hernia sac reduction step.

#### 3.3.2 Ophthalmology

In ophthalmic surgery, Yu et al.^23^ evaluated different machine learning algorithms, including a support vector machine and a CNN‐RNN, to classify surgical phases in cataract surgery. The CNN‐RNN model outperformed others, achieving an AUC of 0.752 for image‐only data, showing high specificity but variable sensitivity across phases.

#### 3.3.3 Orthopedics and thoracic surgery

In orthopedic surgery, Kang et al.^8^ utilized YOLOv3 to identify surgical tools and phases during total hip arthroplasty, achieving a mean average precision (mAP) of 0.7 or higher. For thoracic surgery, Takeuchi et al.^15^ employed a temporal convolutional network (TeCNO) to recognize phases during robot‐assisted minimally invasive esophagectomy (RAMIE), achieving an overall accuracy of 84%.

### 3.4 Anatomical structure recognition

Four studies in this review focused on the recognition of anatomical structures using AI techniques. Jearanai et al.^27^ integrated a deep learning model with an alarm system to precisely detect abdominal wall layers during trocar placement in laparoscopic procedures. Similarly, Smithmaitrie et al.^25^ developed a deep learning framework to assist surgeons in identifying anatomical landmarks in real time during laparoscopic cholecystectomy, aiming to enhance surgical precision and reduce the risk of errors. Mascagni et al.^28^ trained a segmentation model, DEEP‐CVS, to highlight hepatocystic anatomy and assess the achievement of critical view of safety (CVS) criteria during laparoscopic cholecystectomy. Lastly, Une et al.^26^ applied two AI models to recognize hepatic veins and Glissonean pedicle during liver resection, demonstrating the potential of AI for real‐time anatomical navigation during liver surgery (Table 3).

**TABLE 3:** Characteristic of studies focusing on anatomical structure recognition.

Study, year	Type of study	Country	Type of surgery	Dataset	Equipment	Application	Comparator
Smithmaitrie, 2024	OBS	Thailand	General surgery	Proprietary	NA	Anatomical structure recognition	Manual annotation
Une, 2024	OBS	Japan	General surgery	ImageNet datasets	VISERA ELITE II system or an IMAGE1 S™ Camera system for video recording Nu‐VAT as annotation tool for semantic segmentation NVIDIATesla T4 GPU	Anatomical structure recognition	Manual annotation
Jearanai, 2023	OBS	Thailand	Endoscopic surgery	Google Colab platform	NVIDIA A100 Roboflow web application for annotation process	Anatomical structure recognition	Manual annotation
Mascagni, 2022	OBS	France	General surgery	Proprietary: CVS dataset	Open‐source software Pixel Annotation Tool	Anatomical structure recognition	Manual annotation

Abbreviations: AAOS, American Academy of Orthopedic Surgeons; AI, artificial intelligence; CNN, convolutional neural network; CV, computer vision; CVS, critical view of safety; GPU, graphic processing unit; HMM, hidden Markov models; LSTM, long short‐term memory; ML, machine learning; MS‐TCN, multi‐stage – temporal convolutional network; OBS, observational study; REV, review article; RNN, recurrent neural network; SVV, support vector machine; USA, United States of America; VTN, video transformer network.

### 3.5 Performance metrics and AI model comparison

Across the studies, the performance metrics primarily included accuracy, precision, recall, F1 score, and mAP as reassumed in Tables S1 and S2. Ward et al.^22^ compared various models for phase recognition during peroral endoscopic myotomy (POEM), reporting an overall accuracy of 87.6%, with the best performance observed in longer surgical phases. Hashimoto et al.^18^ showed that adding a temporal model to a visual model (ResNet18) improved phase classification accuracy from 82% to 85.6%. Golany et al.^12^ achieved 89% accuracy in recognizing surgical phases of laparoscopic cholecystectomy using a CNN‐ResNet50 with MS‐TCN.

## 4 DISCUSSION

This review highlights the application of AI in various surgical specialties, with a focus on the use of machine learning models for recognizing surgical phases and anatomical structures. Performance comparisons across studies further underscore the consistency of AI models in surgical‐phase recognition, with accuracy ranging from 81% to 93.2%, depending on the procedure. Regarding anatomical structures recognition, models demonstrated good results, with accuracy ranging from 71.4% to 98.1%.

These findings underscore the growing potential of AI in the development of a data‐driven approach in the surgical field. Data extraction and modeling is an essential keys for the development of automated skill assessment, real‐time surgical guidance, and prevention of intraoperative complications.

Readers should critically assess the limitations of the studies presented in this review. To facilitate a deeper understanding, we have included a table summarizing the technical characteristics of the models and experimental designs (Table S3).

Some critics may claim that the majority of included studies utilized internal and proprietary pre‐training datasets; therefore, possibly creating a bias in terms of technique and preferences since videos are collected from the same medical center and surgeries are performed by the same surgeon. This aspect could impact the performance of the AI model when faced with less familiar techniques. However, it must be noted that, among included studies, 6 of them used ImageNet: a vast, globally sourced dataset of visual data primarily used for training AI models, particularly for image recognition, which has seen contributions from researchers, developers, and institutions worldwide.

Enhancing deep learning models by the creation of large international datasets of surgical videos is the key to overpass this current limitation.

Despite the majority of included studies having an overall low risk of bias, moderate risk of bias identified in several studies may influence overall conclusions. Nevertheless, a major strength of our work lies in the correct methodology in the collection and analysis of studies that were followed to prepare this text.

Recent advancements in data science, particularly in machine learning, have reshaped how experts envision the future of surgery. Surgical Data Science is a new field of research that aims to improve the quality of interventional healthcare through the capture, organization, analysis, and modeling of data. While an increasing number of data‐driven approaches and clinical applications have been studied in the fields of radiological and clinical data science, there is still much to improve in the surgical field.^29^

One of the major challenges associated with AI is managing the vast amount of digital data generated during the development and training of algorithms. Big data presents several critical issues, such as storage, classification, and data extraction. Traditional systems struggle to handle this volume of digital data, requiring more efficient solutions like cloud‐based storage. Video indexing, particularly in laparoscopic surgeries, is another challenge, as manually tagging large volumes of video data is time‐consuming and prone to errors. Automated indexing systems are needed to organize data based on relevant content, such as anatomical structures or surgical steps. In this context, data annotation is fundamental as it refers to the process of labeling or tagging data with relevant information that can be used to train AI models. This process is particularly crucial in supervised learning, where an algorithm learns to identify patterns by training on labeled data. Finally, extracting meaningful information from large, unstructured datasets, such as medical images, is complex but essential for accurate AI analysis.

Our review focuses on the progress of deep learning in intraoperative video recording and the automated analysis of surgical phases, anatomical landmarks, and instruments. These tasks are essential for other domains of machine learning, including storage, classification, and data extraction. There are two main approaches to classifying surgical procedure phases using deep learning: content‐based video retrieval, which matches videos to similar ones in the dataset, and segmentation, which breaks down a video into phases and labels each segment. Deep learning, especially multilayer neural networks or artificial neurons, has become essential for recognition tasks due to its success. Unlike traditional models that rely on hand‐crafted features (colors, corners, edges), deep neural networks autonomously learn features from raw data. To be effective, deep convolutional networks need large datasets and a robust methodology covering dataset construction, annotation, training, validation, and testing.^3^

To address this challenge, there are two key strategies: pre‐training on large datasets and data augmentation. The former uses publicly available datasets such as Cholec120,^30^ Cholec80,^31^ and EndoNet^32^ to pre‐train the neural network architecture. The latter enhances the dataset by employing augmentation techniques to increase its size and diversity. Kitaguchi et al.^16^ propose as augmentation a horizontal flip, a vertical flip, and a random crop. Kang et al.^8^ use histogram equalization, flipping, and rotation on the original captured operation image. Jearanai et al.^27^ describe a mosaic augmentation of 16 batch size (random combination of 4 images into one) and a focal loss to address class imbalance.

All the papers reviewed in this study use a pipeline based on CNNs, often combining a visual model with a temporal model. The CNN extracts key visual features from the images, while the temporal model, typically a LSTM network, captures the temporal dynamics of the activities. Both CNN and LSTM parameters are trained on databases of pre‐annotated cases, where surgical phases have been manually labeled. Segmentation, the process of labeling surgical steps, requires expert operators—usually surgeons—to identify the phase of the procedure in each video fragment. Similarly, annotating anatomical landmarks and devices involves classifying the objects and defining their masks and contours. This segmentation process is resource‐intensive, dependent on specialized surgical and anatomical knowledge, and prone to errors. To overcome these challenges, various approaches have been proposed. Golany et al.^12^ and Ward et al.^22^ introduced an inter‐annotator agreement score to assess consistency in annotation, while other studies suggest a hierarchical annotation process, where junior surgeons provide initial annotations, followed by expert revisions for complex cases.^11^, ^26^ Some studies bypass segmentation entirely; for example, Twinanda et al.^24^ introduced RSDNet, a CNN‐LSTM model that predicts remaining surgical duration through regression, demonstrating better generalization across diverse surgeries and surgeon styles. Yengera et al.^20^ proposed a semi‐supervised learning model, using remaining surgery duration prediction as a self‐supervised pre‐training task, significantly reducing the need for large annotated datasets by implementing end‐to‐end pre‐training of CNN‐LSTM networks on long surgical videos.

The technologies discussed in this review have achieved high accuracy (often exceeding 85%) in segmenting procedures into surgical steps, classifying tools, and identifying anatomical landmarks. A recent meta‐analysis^33^ on deep learning accuracy in surgical workflow analysis compared to predetermined labels/annotations showed a pooled sensitivity of 0.95 and specificity of 0.98, reaching promising results. Interestingly, Roi Anteby et al. raised several concerns regarding currently available data, including: lack of external or cross‐validation, arbitrary thresholds for correct segmentation without proper validation, and insufficient details about ground truth labels and the annotation process. To overcome the risk of poor methodology, enhancing international collaboration is crucial, particularly by promoting the standardization of terminology and the establishment of clear guidelines for reporting techniques, methods, and results. Accurate performance estimates can only be achieved through rigorously designed and meticulously executed studies that minimize bias and are reported in a comprehensive and transparent manner.

Evidence arising from the present systematic review confirms a potential application of AI and deep learning also to minimally invasive gynecological surgery.^34^ At the present date, only a few data are available. A recent study by Levin et al.^35^ used an intelligent system that employed computer vision and AI technology to perform automated step recognition of minimally invasive hysterectomy, finding an accuracy between AI‐based predictions and manual human annotations of 93.1%, with a mean‐unweighted accuracy for each step of 87.6%. Literature will need further confirmation of these promising results.

Other potential applications of AI and video analysis in gynecological surgical practice are currently under study. Augmented reality (AR) works by overlaying digital images (US, MR or CT pre‐operative scan) onto real‐time laparoscopic view of the surgical field, providing surgeons with real‐time 3D visualization of hidden critical anatomical structures. Several studies^36^, ^37^, ^38^ highlighted the applicability of AR software to guide surgery of the uterus, especially in cases of myomectomies in which the correct location of fibroids and key structures improves surgical success and reduces the risk of accidental injury.

Current research is also directed on the creation of datasets of laparoscopic gynecological images that can be used to train artificial neural networks in semantic segmentation, like the one proposed by Madad Zadeh et al.,^2^, ^39^ in which a CNN not only identifies the presence or absence of particular anatomical structures but, more crucially, categorizes each pixel in the image, indicating the anatomical structure it corresponds to. Deep learning semantic segmentation could potentially improve gesture safety during minimally invasive gynecological surgery, representing a promising field of research. By facilitating accurate anatomical recognition, such systems may reduce the likelihood of intraoperative errors and complications. Moreover, this technological advancement paves the way for a broader transformation of surgical practice—particularly in surgical education and skill acquisition. Integration of AI‐guided tools into training protocols will benefit novice surgeons with a more structured and accelerated learning process that combines theoretical instruction with real‐time visual guidance. This may substantially shorten the learning curve required to achieve surgical autonomy.

## 5 CONCLUSION

This systematic review underscores the potential of AI to assist surgeons in phase recognition, tools, and anatomical landmark detection. The reviewed evidence demonstrates that AI models, especially those integrating convolutional and temporal neural networks, have achieved high levels of accuracy in identifying surgical phases, instruments, and anatomical landmarks across various surgical specialties. The automatic indexing of documented procedures can be useful for educational purposes, analysis of complications, and prediction of surgery time, providing feedback for surgical skill development and improving operating room efficiency. Despite methodological heterogeneity and limitations—such as small, non‐generalizable datasets and variability in annotation protocols—these technologies represent a significant step toward data‐driven surgery. Regarding clinical implications, the integration of AI in the operating room could substantially enhance intraoperative safety by supporting real‐time phase recognition and anatomical structure detection, thereby reducing the risk of critical errors. Secondly, these technologies hold promise in revolutionizing surgical education by accelerating the acquisition of procedural knowledge and technical skills. To fully realize the potential of AI in surgery, there is an urgent need for the creation of large, standardized, and representative international datasets. Cross‐disciplinary collaborations and consensus on reporting standards will be key to advancing this field.

## AUTHOR CONTRIBUTIONS

Conceptualization and supervision: AAB, AB, NB, GV; writing—original draft preparation: SP, CT, GP, VT; review and editing: SP, CT, VT; supervision: MT, CB; project administration: AAB, AB, NB, GV. All authors have read and agreed to the published version of the manuscript.

## FUNDING INFORMATION

This research received no external funding.

## CONFLICT OF INTEREST STATEMENT

The authors declare no conflict of interest.

## ETHICS STATEMENT

Since the research has a bibliographic‐retrospective nature, it did not require approval from the local ethics committee.

## Supporting information


Table S1–S3.

Appendix S1.

## Data Availability

All data relevant to the study are included in the article or uploaded as Supporting Information. All data were extracted from previously published studies; thus, they are publicly available.
