# Supplementary material for: Artificial intelligence in the operating room: A systematic review of AI models for surgical phase, instruments and anatomical structure identification
Source: Acta Obstet Gynecol Scand. 2025 Aug 27;104(11):2054–64. doi: 10.1111/aogs.70045 (PMC12575173; doi:10.1111/aogs.70045)
Supplement: Supplementary file 1 — Table S1–S3. [file AOGS-104-2054-s002.docx]

**Table S1. Outcomes of studies focusing on Surgical Phase Recognition**

| **Stud y, year** | **Procedure type** | **Surgical landmarks** | **No of videos** | **Annotators** | **Performance scores** | | | |
| --- | --- | --- | --- | --- | --- | --- | --- | --- |
|  |  |  |  |  | **Accuracy** | **Precision** | **F1**  **s c o r e** | **Others** |
| Kang, | Total Hip | 7: skin | 540  operation images | 1 | mAP |  |  |  |
| 2024 | Arthoplasty | incision;  broaching; |  |  | for  phases |  |  |  |
|  |  | exposure of |  |  | : 0.10- |  |  |  |
|  |  | acetabolum; |  |  | 0.97 |  |  |  |
|  |  | acetabular |  |  |  |  |  |  |
|  |  | reaming; |  |  | mAP |  |  |  |
|  |  | acetabular |  |  | for |  |  |  |
|  |  | cup |  |  | instru |  |  |  |
|  |  | positioning; |  |  | ments: |  |  |  |
|  |  | femoral stem |  |  | 0.21- |  |  |  |
|  |  | insertion; |  |  | 0.97 |  |  |  |
|  |  | skin closure. |  |  |  |  |  |  |
| Kom | Distal |  |  | 3 | 88.8% |  | 0.5 |  |
| atsu,  2024 | Gastrectomy | 9: preparation; | 56 |  |  |  | 9 -  0.9 |  |
|  |  | LND on the left | submitted |  |  |  | 6 |  |
|  |  | greater | to the |  |  |  |  |  |
|  |  | curvature; LND | ESSQS |  |  |  |  |  |
|  |  | on the right |  |  |  |  |  |  |
|  |  | greater |  |  |  |  |  |  |
|  |  | curvature; |  |  |  |  |  |  |
|  |  | transection of |  |  |  |  |  |  |
|  |  | the duodenum; |  |  |  |  |  |  |
|  |  | supra- |  |  |  |  |  |  |
|  |  | pancreatic LND; |  |  |  |  |  |  |
|  |  | LND on the |  |  |  |  |  |  |
|  |  | lesser |  |  |  |  |  |  |
|  |  | curvature; |  |  |  |  |  |  |
|  |  | transection of |  |  |  |  |  |  |
|  |  | the stomach; |  |  |  |  |  |  |
|  |  | reconstruction; |  |  |  |  |  |  |
|  |  | completion of |  |  |  |  |  |  |
|  |  | surgery. |  |  |  |  |  |  |

| Fer 2023 | Gastric Bypass |  | 545 | 11 |  |  | 0.7  8 -  >.9  0 |  |
| --- | --- | --- | --- | --- | --- | --- | --- | --- |
|  |  | 16: access; adhesiolysis; mobilization of stomach;  gastric pouch creation; reinforce gastric pouch staple line; division of omentum; measurement of bowel; preparation of GJS; GJS (linear stapled or hand sewn); GJS  (circular); JD; Preparation of JJS. JS. Closure of mesentery. HHR. Closure. |  |  |  |  |  |  |
| Orte nzi 2023 | Totally Extraperi toneal  Inguinal Hernia Repair | 6: Balloon dissection; Extraperitoneal access (trocars); Preperitoneal dissection; Hernia and sac  reduction; mesh  placement; mesh fixation | 619 | 3 | Overall  : 88.8%  Per- step: 72.2% -  94.3% |  |  |  |
| Gol any 202  2 | Cholecy stectom y | 8: trocar insertion, preparation,  Calot triangle  dissection, clipping and cutting,  gallbladder dissection, gallbladder packaging, cleaning and  hemostasis, gallbladder extraction  4 Adverse events: | 371 | 2 | 89% |  |  | Annotation of the CVS  during the Calot triangle  dissection phase |

|  | major  bleeding, gallbladder perforation, major bile leakage,  and  incidental finding. | |  |  |  |  |  |  |
| --- | --- | --- | --- | --- | --- | --- | --- | --- |
| Kita guc hi, 202  2 | Transanal Total Mesorectal Excision | 5:PSC; full thickness  transection of the rectal wall; down- to-up  dissection; dissection after  rendezvous  ; PSC for stapled anastomosi s. | 50 | 2 | overal l 93.2% |  | 0.  71  -  0.  99 |  |
| Sasaki 2022 | Hepatectomy | 9:  Extracorporeal action; port  insertion; adhesiotom y; marking resection  line;  encircling of hepatoduod enal  ligaments and hepatic inflow-  occlusion; mobilization of the liver; liver  transection; specimen extraction; closing of the surgical incision. | 40. 8  million | 4 | Model 1:  0.891  Model 2:  0.947  Media n accur acy for each case  in  Model  2 was  0.927  (range  , 0.884–  0.997)  and |  |  |  |

|  |  | |  |  | 0.937  ± 0.04. |  |  |  |
| --- | --- | --- | --- | --- | --- | --- | --- | --- |
| Tak euc hi, 202  2 | Esophagectomy | 8:  preparation, PFI; PFD,  hernia  dissection, mesh  deployment  , mesh  fixation, peritoneal  flap closure, additional  closure | 31 | 2 | Overal l 84%. |  |  |  |
| Tak euc hi 202  2 | Inguinal Ernia Repair | 7:  preparation, peritoneal fap incision, PFI, hernia dissection, mesh  deployment  , mesh  fixation,  closure of peritoneal fap,  additional closure | 119 | 1 | 88.8%  (unilateral); 85.8  %  (bilateral). |  |  |  |
| Ward 2021 | Peroral Endoscopic Myotomy | 5: SI,  Mucosotomy, ST Myotomy, MC. | 50 | 3 | Overal l 87.6%.  For  phase s: 70.6%  that had a duration |  |  |  |

|  |  | |  |  | under five minut  es and 88.3%  for  longer phase s. |  |  |  |
| --- | --- | --- | --- | --- | --- | --- | --- | --- |
| Kita guc hi  202  0 | Colorectal surgery |  | 300 | 2 | Accuracy for  autom atic SPR: 81.0%;  Accuracy for action  classification: 83.2%. |  | 0-  68 –  0.  88 | Mean IoU for the  autom atic  tool segmentation task for 5 tools was  51.2%  IoU Tool 1-5:  33.6%-68.9%-65.6%-52.6%-35.3%  . |
|  |  | 9: TME (right side); Medial mobilization of colon;  Transection of IMA; Medial mobilization of colon; Lateral mobilization of colon; TME  (left side); Dissection of mesorectum; Transection of rectum and anastomosis. Transection of IMV/LCA. |  |  |  |  |  |  |
| Has him oto, 201  9 | Sleeve Gastrec tomy | 7: port  placement, liver retraction,  liver biopsy, gastrocolic ligament  dissection, stapling of the stomach, bagging specimen,  final  inspection | 88 | 2 | Mean (±SD)  accuracy in identifying  operat ive steps: 82% ±  4%  (visual model 81.9%,  sleeve  Net |  |  | MCC  coefficient for  human  annotators: 0.862. |

|  | of staple line. |  |  | 73-  85.6%) |  |  |  |
| --- | --- | --- | --- | --- | --- | --- | --- |
| Kita guc hi  201  9 | Sigmoidectomy | 71 | 19 | Overal l accuracy of the Phase 1–9  model  : 90.1%  Phase I–VII  model  : 91.9%.  Recognition accuracy is 85.2%. |  |  |  |
|  | 11:  Preparation, TME, Medial mobilization of colon,  transection of IMA, lateral mobilization of colon, TME, dissection of mesorectum,  transection of rectum and anastomosis,  transection of IMV, after anastomosis  2 actions: Extracorporea l actions and intracorporeal (i.e.  irrigation). |  |  |  |  |  |  |
| Pad oy 201  9 | Cholecystectomy | 120 | 33 | Recognition accuracy over 90%. |  |  |  |
|  | Cholec120, 6: Calot triangle dissection;  Clipping- Cutting;  Gallbladder dissection;  Packaging; Cleaning/coagulation, extraction |  |  |  |  |  |  |

|  |  | |  |  |  |  |  |  |
| --- | --- | --- | --- | --- | --- | --- | --- | --- |
| Twi nan da, 201  9 | Cholecystectomy  Gastric Bypass | Cholec80 dataset, 7:  preparation;  Calot triangle dissection;  clipping and cutting;  gallbladder dissection; packaging;  cleaning and coagulation; gallbladder retraction.  ENDO-vis, 7:  placement trocars, preparation,  clipping and cutting;  gallbladder dissection; retrieving gallbladder, hemostasis,  drainage and closing. | 120 for cholecistectom y; 170  for gastric bypass | 33 |  |  |  | MAE  in  minute for RDS  estimation. |

| Yu, 201  9 | Cataract | 10: side incision, main  incision, capsulorrhexis,  hydrodissection,  phacoemulsification, cortical removal, lens  insertion, ophthalmic  viscosurgical device removal,  wound closure (corneal  hydration) | 100 | 1 |  | Precision: 0.28  3 to  0.96  3. |  | AUC  for the image  -only CNN- RNN (0.752  ) was greater than that of the CNN  with cross- sectional image data. Specificity  high for all phase s.  Sensitivity: 0.005 (95%C  I) to 0.015  for the suppo rt vector machine for wound  closure (corne al hydration) and 0.974  for the RNN  for main incision. |
| --- | --- | --- | --- | --- | --- | --- | --- | --- |

| Yenge ra,  2018 | Cholecystectomy | 7: Preparation; calot triangle dissection;  clipping & cutting;  gallbladder dissection; gallbladder packaging;  cleaning & coagulation; gallbladder extraction. | Cholec- 120 | 33 |  | F1  scor e 81.1  +/- 7.5. |  |  |
| --- | --- | --- | --- | --- | --- | --- | --- | --- |

THA: total hip arthroplasty; DL: deep learning; LND: lymphadenectomy; ESSQS: Endoscopic Surgical SKill Qualification System; SPR: surgical phase recognition; P3: phase “n”; AICS: AI confidence score; PR: phase recognition; DC: Dice coefficient; LLR: laparoscopic liver resection; GJS: Gastrojejunostomy; JJS: jejunojejunostomy; JS: jejunostomy; HHR: Hiatal hernia repair; IOP: intraoperative; AW: abdominal wall; ARS: anterior rectus sheath; RM: rectus muscle, PRS: posterior rectus sheath; TEP: laparoscopic totally extraperitoneal inguinal hernia repair; CVS: Critical View of Safety; PSC: purse-string closure; SD: standard deviation; TaTME: Transanal Total Mesorectal Excision; LH: laparoscopic hepatectomy; PFI: peritoneal flap incision; PFD: peritoneal flap dissection; RAMIE: robot-assisted minimally invasive esophagectomy; POEM: peroral endoscopic myotomy; PFI: peritoneal fap dissection; UH: unilateral hernia; ST: Submucosal tunnel, SI: Submucosal injection; MC: Mucosotomy closure; TME: Total mesorectal excision; IMA: inferior mesenteric artery; IMV: Inferior mesenteric vein; LCA: left colic artery; CV: computer vision; MCC: Mean concordance correlation; CfT: convergence for training; MAE: Mean absolute error; AUC: area under the receiver operating characteristic curve; ML: machine learning.

**Table S2. Outcomes of studies focusing on Anatomical Structure Recognition.**

| **Study,**  **year** | **Procedure type** | **Surgical**  **landmarks** | **No of videos** | **Annotators** | **Performance scores** | | | | | | | | |  |
| --- | --- | --- | --- | --- | --- | --- | --- | --- | --- | --- | --- | --- | --- | --- |
|  |  |  |  |  | **Accuracy** | **Precision** | | | | **F1 score** | | **Others** | |  |
| Smithmaitrie, 2024 | Cholecystectomy | 7: liver base of segment IV; Rouviere’s sulcus, slit type; Rouviere’s sulcus; open type; Rouviere’s sulcus, scar type. | 40 | 3 | mAP 98.1%. | |  | | | . | | Dissection line: 95.7% acceptance by surgeons.  Comparative analysis of YOLO v7 with YOLO v4 and v5 demonstrate superiority of YOLO v7. | |  |
| Une, 2024 | Liver Resection | 2: hepatic vein and Glissonean pedicles | 53 | 4 |  | |  | | |  | | Average DC for the 2-class model: 0.789 ± 0.008; 3-class model: for hepatic vein 0.631 ± 0.009, for Glissonean pedicles 0.482 ± 0.055. | |  |
| Jearanai, 2023 | Trocar insertion with optical trocar | 6 of AW: subcutaneous, ARS; RM; PRS, peritoneum, and abdominal cavity. | 89 | 3 | Accuracy (mAP): 95.8%; | | precision: 89.8%; | | |  | | recall: 91.7%.  In real time experiment, the trained model detected each layer of the abdominal wall. | |  |
| Mascagni, 2022 | Cholecystectomy | 7 of hepatocystic anatomy: gallbladder, cystic duct, cystic artery, dissected hepatocystic triangle, cystic plate, surgical tools and background. | 201 videos + 402 images | 2 | Adverse events: accuracy 71.4%. | Adverse events: average precision 71.9%; | | | |  | | Anatomical landmarks: mean Intersection Over Union (IoU): 66.6%. | |  |
|  |  |  |  |  |  |  | |  |  | |  | |  | |

mAP: mean average precision; DC: Dice coefficient; AW: abdominal wall; ARS: anterior rectus sheath; RM: rectus muscle; PRS: posterior rectus sheath; DaNN: deep artificial neural network; DeepCVS: Critical View of Safety.

**Table S3. Technical characteristics of models**

| **Study, year** | **Algorithm (baseline architecture) ^a^** | **Augmentation** | **Pre-training** | **Database for pre-training** | **Supplementary prediction models ^b^** | **Experiment design** |
| --- | --- | --- | --- | --- | --- | --- |
| Kang, 2024 | YOLOv3 | histogram equalization, flipping, rotation to the original capture operation image. | N | NA | N | 330 images of surgical instruments and 210 images of surgical procedures.  Cross-section of labels with a unique combination of instruments used during the phase |
| Komatsu, 2024 | EfficientNetB7 | N | N | N | N | Random training and validation sets (90% vs 10% )  hold-out validation performed |
| Fer, 2023 | ResNet R(2+1)D  MS-TCN | Transfer learning | Y | Large free datasets ^c^ | MS-TCN | 390 videos for training, 95 for validation, 60 for testing. |
| Ortenzi, 2023 | VTN | Transfer learning | Y | Multiple general procedures | LSTM network | 371 videos for training, 93 for internal validation, 155 as a test set. |
| Golany, 2022 | Resnet50 | N | N | Cholec80 | feature extraction model; temporal aggregation model with MS-TCN | Split 80:20 ratio for training and testing  on a per-case rather than a per-frame level |
| Kitaguchi, 2022 | Xception | N | Y | ImageNet | N | Split 80:20 ratio for training and testing  on a per-case rather than a per-frame level.  Cross-validation not performed. |
| Sasaki, 2022 | Xception | N | N | ImageNet | NA | 30 and 10 videos were randomly assigned to a training dataset and test dataset. |
| Takeuchi, 2022 | TeCNO | N | N | N | MS-TCN | k=4 folds cross validation, random set of 75% of the videos for training and the remaining 25% for testing. |
| Takeuchi 2022 | TeCNO | N | N | N | HMM | NA ^c^ |
| Ward, 2021 | ResNET | N | Y | PyTorch Library | LSTM network  HMM | Random training on 30 videos.  Test on the remaining 20 videos. |
| Kitaguchi, 2020 | Xception | Horizontal flip, vertical flip, random crop | Y | ImageNet | Semantic segmentation by U-Net model | Training subset contained 66, 001, 529 annotated images (from 240 videos); test subset contained 16, 621, 569 annotated images (from the remaining 60 videos) |
| Hashimoto, 2019 | ResNet 18 | N | Y | ImageNet 2012 | LSTM network | Split 70:30 ratio for training and testing  on a per-case level rather than at a per-frame level. |
| Kitaguchi 2019 | Inception- Res-Net-2 | N | Y | Keras | LSTM network  light gradient boosting machine (LightGBM) | A total of 7,832,652 frames were extracted from 63 cases, and used as the training data, whereas 927,417 frames were extracted from 8 cases, and used as the test data. |
| Padoy ,2019 | CNN-NMM | NA | NA | NA | LSTM network | NA |
| Twinanda, 2019 | RDSNet (ResNet152) | Finetune with a batch size of 48 | Y | ImageNet  Cholec120  Bypass170 | LSTM network | The LSTM network is trained for a total of 30k iterations on the Cholec120 dataset and 50k iterations on the Bypass170 dataset, with an initial learning rate of 10−3.  After every 10k iterations on the Cholec120 dataset and 5k iterations on the Bypass170 dataset the learning rate is decayed by a factor of 10.  A weight decay parameter of 10−2 is utilized. |
| Yu, 2019 | RNN | NA | NA | NA | LSTM | 5-fold cross-validation  Training/Testing 3:1 |
| Yengera, 2018 | EndoN2N N Y | | | Self-supervised RDS pre-training model | LSTM network: EndoLSTM | End-to-end training approach by approximation of the BPTT algorithm ^c^. |
| Smithmaitrie, 2024 | YOLOv7 Mosaic method N | | | NA | guided dissection line algorithm  E-ELAN | From the total collection of 3200 images, the dataset was divided into three subsets: 2240 images for training, 320 for validation and 640 for testing. |
| Une, 2024 | EfficientNetV2 N Y | | | ImageNet | NA | Training/ test 5:1 ratio on a per-case rather than on a per-frame basis |
| Jearanai, 2023 | YOLOv8 Mosaic model N | | | NA | NA | 3600 images separatedinto three subsets: a training set of 3000 images, a validationset of 200 images, and a test set of 400 images. |
| Mascagni, 2022 | DEEP-CVS:  2 stage model Transfer learning Y | | | pretrained on a large dataset | NA | 5-fold cross-validation was performed.  3 folds (60%) for training, 1 for validation (20%), and 1 for testing (20%).  The validation set was used to pick model hyperparameters  and determine when to stop training. |

^a^ Best performing CNN model when more than one model was tested

^b^ Deep-learning-based features or other ML model to improve the baseline CNN

^c^ More information in the paper

LSTM: Long-Short term memory networks; HMM: Hidden Markov model; SVM: Support Vector Machine; RDS: remaining surgery duration; MS-TCN: Multi-stage temporal convolutional networks; VTN: Video Transformer Network; TeCNO: Temporal Convolutional Networks for the Operating room; RNN: Recurrent Neural Network; E-ELAN: Extended Efficient Layer Aggregation Networks.
