# Supplementary material for: Artificial intelligence in the operating room: A systematic review of AI models for surgical phase, instruments and anatomical structure identification
Source: Acta Obstet Gynecol Scand. 2025 Aug 27;104(11):2054–64. doi: 10.1111/aogs.70045 (PMC12575173; doi:10.1111/aogs.70045)
Supplement: Supplementary file 2 — Appendix S1. [file AOGS-104-2054-s001.docx]

**Supporting Information**

**Appendix S1. Search Strategy**

Pubmed

(("segmentation"[All Fields] OR "contouring"[All Fields]) AND "Artificial Intelligence"[All Fields] AND "deep learning"[All Fields] AND "laparoscop1"[All Fields] AND "model"[All Fields]) NOT ("diagnostic imaging"[MeSH Subheading] OR ("diagnostic"[All Fields] AND "imaging"[All Fields]) OR "diagnostic imaging"[All Fields] OR "ultrasound"[All Fields] OR "ultrasonography"[MeSH Terms] OR "ultrasonography"[All Fields] OR "ultrasonics"[MeSH Terms] OR "ultrasonics"[All Fields] OR "ultrasounds"[All Fields] OR "ultrasound s"[All Fields])

Translations

ultrasound: "diagnostic imaging"[Subheading] OR ("diagnostic"[All Fields] AND "imaging"[All Fields]) OR "diagnostic imaging"[All Fields] OR "ultrasound"[All Fields] OR "ultrasonography"[MeSH Terms] OR "ultrasonography"[All Fields] OR "ultrasonics"[MeSH Terms] OR "ultrasonics"[All Fields] OR "ultrasounds"[All Fields] OR "ultrasound's"[All Fields]

Web of Science

TS= (("segmentation" OR "contouring")

AND "Artificial Intelligence"

AND "deep learning"

AND "laparoscop1"

AND "model")

NOT TS=("diagnostic imaging" OR ("diagnostic" AND "imaging") OR "ultrasound" OR "ultrasonography" OR "ultrasonics" OR "ultrasounds")

EBSCO

TX= ("segmentation" OR "contouring")

AND TX="Artificial Intelligence"

AND TX="deep learning"

AND TX="laparoscop1"

AND TX="model"

NOT TX=("diagnostic imaging" OR ("diagnostic" AND "imaging") OR "ultrasound" OR "ultrasonography" OR "ultrasonics" OR "ultrasounds")
